# Commentary: Constructing the optimal experimental autoimmune thyroiditis mouse model using porcine thyroglobulin

**DOI:** 10.3389/fimmu.2025.1690618

**Published:** 2025-12-01

**Authors:** Min Li, Yingchun Zhou, Ming Cai

**Affiliations:** Department of Thyroid Oncology, Chongqing Key Laboratory of Translational Research for Cancer Metastasis and Individualized Treatment, Chongqing University Cancer Hospital, Chongqing, China

**Keywords:** molecular mechanism, animal model construction, NOD/LtJ mice, experimental autoimmune thyroiditis, autoimmune thyroiditis

## Introduction

The study by Liu et al., titled “Constructing the optimal experimental autoimmune thyroiditis mouse model using porcine thyroglobulin,” published in *Frontiers in Immunology*, provides a systematic evaluation of immunization strategies for inducing autoimmune thyroiditis (AIT) in NOD/LtJ mice (1). By comparing different antigen doses, immunization frequencies, and injection routes, the authors identify a high-dose (200 µg pTg) and high-frequency (three immunizations) protocol as optimal for modeling AIT, with tail vein injection favoring antibody production and subcutaneous injection promoting stronger histological inflammation. This work offers valuable practical guidance for researchers aiming to establish reproducible AIT models. However, several methodological and translational aspects merit further considerations.

To fully contextualize the work by Liu et al., it is important to consider the historical development of EAT models. The foundational studies, notably by Dr. Noel Rose and colleagues, successfully induced thyroiditis using heterologous thyroglobulin, a strategy that Liu et al. now seek to optimize (2). A significant subsequent advancement came from the work of Dr. Yi-chi Kong’s lab, which emphasized the critical role of self-antigen (murine thyroglobulin) in conjunction with adjuvant to break tolerance, more closely mimicking the breach of self-tolerance in human disease (3–5). Furthermore, it is established that regulatory T cells (Tregs) are pivotal in maintaining tolerance, as their depletion can trigger autoimmune thyroiditis even without adjuvant. The genetic underpinnings of AIT are also well-documented; in humans, specific HLA haplotypes confer susceptibility, which is reflected in mouse models through the use of the H-2K haplotype (6). Acknowledging this rich historical and mechanistic landscape allows for a more nuanced appreciation of the model presented by Liu et al. and its position within the ongoing quest to recapitulate human AIT.

## Subsections relevant for the subject

First, the use of NOD/LtJ mice as an alternative to the less accessible NOD.H-2h4 strain is pragmatically justified and enhances model accessibility. However, the genetic and immunological differences between these strains—particularly in MHC haplotype and spontaneous vs. induced disease onset—may influence translational relevance to human AIT. Future studies should include comparative transcriptomic or proteomic analyses to clarify strain-specific immune phenotypes and their alignment with human disease (7, 8).

Second, the comprehensive multi-parameter assessment—including histopathology, serum antibodies, cytokines, and local immune cell infiltration—strengthens the model’s validity. The incorporation of multiplex immunofluorescence and immunohistochemistry for Th17/Treg balance and inflammasome markers (NLRP3, Caspase-1) is particularly commendable. Nevertheless, the absence of B-cell and follicular helper T-cell (Tfh) analysis represents a significant opportunity for deeper investigation. As the reviewer rightly highlights, this is a critical aspect. Elaborating further, B cells are not only precursors to autoantibody-producing plasma cells but also function as antigen-presenting cells and regulators of T-cell responses in AIT. Similarly, Tfh cells, located in B-cell follicles, are specialized in providing help for B-cell affinity maturation and antibody class switching. Their coordinated action is pivotal for the development of tertiary lymphoid structures often observed in chronic autoimmune thyroiditis. Therefore, quantifying B-cell and Tfh infiltration and their spatial organization within the thyroid would substantially enhance our understanding of the humoral immune mechanisms at play in this model (9, 10). Furthermore, enhancing the histopathological analysis would strengthen the model’s characterization. The study’s iconography primarily provides low-magnification overviews of thyroid inflammation. While useful for assessing the overall inflammatory area, higher-magnification images are crucial for two key reasons: first, to better characterize the specific types of immune cells within the infiltrate, and second, to reliably distinguish genuine inflammatory foci from ectopic thymic tissue, a known histological feature in NOD mice that can be mistaken for lymphocytic infiltration. Such detailed microscopy would provide more definitive evidence of autoimmune pathogenesis and improve the accuracy of histological scoring.

Third, the study highlights the superiority of triple immunization over double immunization in inducing severe thyroiditis, which aligns with immune memory principles. We agree with the authors’ own recognition that the lack of longitudinal tracking beyond the acute phase (4 weeks post-immunization) is a limitation of their study. As our commentary and the reviewers of the original article suggest, this is a highly relevant point for the field. Extending the observation period to include time-series assessments at 8–12 weeks would be invaluable to model the chronicity of human AIT, monitor potential disease progression or remission, and ultimately allow for the evaluation of lasting therapeutic interventions (11).

Fourth, while the tail vein method enhanced antibody production and NLRP3 activation, its slightly lower inflammation scores compared to subcutaneous injection suggest route-dependent immune polarization. This observed route-dependent immune polarization indeed merits deeper mechanistic inquiry. To truly dissect the underlying mechanisms, future studies could employ techniques such as *in vivo* cell tracking of adoptively transferred antigen-pulsed dendritic cells to compare their trafficking to the spleen (systemic immunity) versus draining lymph nodes (local immunity) following IV or SC injection. Additionally, detailed immunophenotyping of the resulting immune responses in these lymphoid organs and the thyroid itself could reveal differences in T-cell polarization, germinal center formation, and the establishment of local versus systemic immune memory, providing a clearer rationale for selecting one injection route over the other based on the specific research objectives. Additionally, the use of LPS in the IV protocol may introduce systemic inflammation confounding thyroid-specific responses (12).

Fifth, the study appropriately acknowledges the sex-specific limitation of using an all-female cohort. As the reviewer notes, this is a useful starting point for further investigation. Future work should systematically compare both male and female mice to elucidate sex differences in immune response kinetics and disease severity. This approach could reveal crucial hormonal or genetic modifiers of AIT. Furthermore, integrating sex as a biological variable into more complex models, such as those combining genetic modifications with environmental factors like iodine supplementation, could powerfully recapitulate the heterogeneity seen in the human AIT patient population (13).

## Discussion

This study delivers a rigorously optimized protocol for inducing AIT in NOD/LtJ mice, balancing pathological severity with operational feasibility. The integration of immunological and histopathological endpoints provides a robust framework for model validation. However, the translational impact would be strengthened by including human thyroid tissue validations, extending observation to chronic phases, and incorporating B-cell and Tfh analyses. Furthermore, exploring combinatorial models—e.g., by integrating genetic predispositions with environmental triggers like iodine supplementation and by considering sex as a key biological variable—could better recapitulate the complex heterogeneity of human AIT. By providing a rigorously optimized protocol within the established framework of heterologous antigen-induced EAT, Liu et al. offer a valuable and accessible resource for accelerating preclinical research in autoimmune thyroiditis.
